# Evaluation of novel nanoscaled metal fluorides on their ability to remineralize enamel caries lesions

**DOI:** 10.1038/s41598-018-38225-8

**Published:** 2019-02-13

**Authors:** Matthias Zirk, Sandra Schievelkamp, Erhard Kemnitz, Julian Lausch, Richard J. Wierichs, Marcella Esteves-Oliveira, Hendrik Meyer-Lueckel

**Affiliations:** 1 Department for Oral and Craniomaxillofacial and Plastic Surgery, University of Cologne, Cologne, Germany; 2 Department of Operative Dentistry, Periodontology and Preventive Dentistry, RWTH Aachen University, Aachen, Germany; 3 Department for Chemistry, Humboldt-Universität of Berlin, Berlin, Germany; 4 Department of Biohybrid & Medical Textiles (BioTex), AME - Institute of Applied Medical Engineering, Helmholtz Institute, RWTH Aachen University, Aachen, Germany; 5 Department of Restorative, Preventive & Pediatric Dentistry, Universität Bern, Bern, Switzerland

## Abstract

The aim of this *in vitro* study was to evaluate the ability of two nanoscaled metal fluorides (NMF) to remineralize shallow (SL) and deep (DL) artificial enamel caries lesions. NMF are synthesized via a non-aqueous fluorolytic sol-gel-synthesis and dissolve low fluoride concentration in aqueous solutions (n-CaF_2_: 7 ppm, n-MgF_2_: 70 ppm), whilst containing a nominal fluoride concentration of 3,400 ppm. For comparison, an aqueous sodium fluoride solution (NaF: 3,400 ppm), a sodium fluoride containing varnish (Duraphat: 22,600 ppm) and a fluoride-free negative control were investigated. Bovine enamel specimens with SL (n = 86, 4649–4795 vol%xμm) or DL (n = 145, 9091–9304 vol%xμm) were prepared and allocated to five groups each. In each group the respective agent was applied and pH-cycling was performed for 14 days (SL) and 90 days (DL), respectively. Mineral loss and lesion depth were assessed by transversal microradiography. For SL, all fluoride agents significantly remineralized the specimens compared to baseline (p > 0.05; Mann-Whitney test) to a similar extent. For DL, both NMF showed significantly higher mineral gain compared to the other fluoride agents (p < 0.05). In conclusion, the novel NMF- showing relatively low free fluoride concentrations- bear at least the similar potential for remineralization of early caries lesions as highly fluoridated agents being commonly used.

## Introduction

Topical fluorides are commonly applied to prevent caries formation^1,2^. Sodium fluoride (NaF) is the most frequently used fluoride compound^1^. Different modes of fluoride application (e.g. toothpastes, gels, varnishes and mouth rinses) are widely documented in the literature including their dose dependent therapeutic effect^2–4^. Immediately after fluoride application, rather high concentrations of fluorides are detectable in oral fluids and plaque. However, a few minutes after application the fluoride concentration decreases distinctly^5^. Therefore, frequent applications of rather low concentrations of fluorides are recommended to inhibit demineralization and enhance remineralization^5^. The tongue may serve as an important fluoride reservoir, as well^6^. For remineralisation *in vivo*, the oral fluoride bioavailability depends of at least four factors: fluoride formulation, time after fluoride application, fluoride concentration in supernatant saliva and fluoride concentration in salivary sediment^7^.

The process of remineralization of enamel caries lesions is limited by the presence of calcium and phosphate ions from an external source to promote the deposition of ions into the enamel structure^8^. Fluoride-containing varnishes (e.g. Duraphat) are applied to promote long-term remineralization by creating loosely bounded calcium-fluoride reservoirs^9^. However, these slow fluorides releasing CaF_2_-like reservoirs are not considered to be effective under higher cariogenic conditions^10^. Thus, their influence on remineralization is somehow limited to directly accessible dental surfaces^10^. In enamel, calcium ions and hydroxyl groups of hydroxyapatite (HA, Ca_5_(PO_4_)_3_(OH)) are frequently replaced by sodium (Na^+^) and magnesium (Mg^2+^) or fluoride (F^−^), carbonate (CO^2−^) as well as chlorine (Cl^−^), respectively^4^. Between the dense and highly mineralized hydroxyapatite structure of the enamel the intercrystalline space offers a pathway for ions in order to permeate into the enamel^11^. Sound enamel showed permeability parameters of 0.22 m^2^ g^−1^ in area, 2.8 mm^3^ g^−1^ in pore volume and 22 nm in pore size^12^. Recently, some engineered nanomaterials were developed in order to penetrate into the mineral structure, but their capability of remineralizing the dental enamel structure has not been fully elucidated, yet^4^.

Several approaches using nano-like materials have been reported. In some studies, caries lesions were treated *in vitro* with biomimetic enamel-like nanomaterials^13^. Carbonate-hydroxyapatite nanocrystals in size of 20 to 100 nm presented a capability of depositing into erosively altered enamel and progressively refilling surficial scratches of the enamel^14^. Furthermore, nano-HA particles smaller than 20 nm were able to integrate into the surface matrix of enamel, regardless of their different hierarchical structure^15^. In general, artificial nano-HA is produced in spherical, needle-like and crystalline forms^4^. Nevertheless, artificial nano-HA in sizes smaller than 20 nm seem to be more instable than 40 to 80 nm sized HA particles^4,15^. The formation of a fluoridated or fluoride-free biomimetic enamel-like layer containing nano-sized apatite crystals as a result of a self-organized process induced by the extracellular matrix protein amelogenin has been described as a feasible method for remineralizing enamel in laboratory studies^16,17^.

Another approach is the use of casein phosphopeptide (CPP) produced by tryptic digest of the milk protein casein, in order to form nano-clusters of amorphous calcium phosphate (ACP) that reduce demineralization and promote remineralization^18^. Subsequently, free calcium and phosphate ions leave the CPP-ACP and reform onto apatite crystals^19^. Furthermore, ACP dissociates to calcium and phosphate ions to offset pH-dropping in the plaque as a buffer, thereby minimizing enamel demineralization^19^. In experiments, creation of nano-calcium fluoride particles by a spray-drying method with Ca(OH)_2_ and NH_4_F solutions displayed a valuable result regarding remineralization in *vitro*^20,21^. Hereby, it was intended to produce labile fluoride reservoirs for continuous remineralization^20,21^. In summary, the application of engineered nanomaterials integrated into dentifrices to improve oral health and prevent caries are described using various methods^4,13^. The current regulatory procedures of how to apply engineered nanomaterials are still part of the recent discussion^4^. The great diversity in nanomaterials is constituted by the different types of productions creating variable capabilities for newly developed nanomaterials^4,13^. Novel nanomaterials are recommended to be tested *in vitro* before starting further clinical investigations^22^.

Therefore, the present *in vitro* study was conducted to evaluate the influence of two novel nanoscaled metal fluorides (NMF) on caries lesions in enamel. These NMF were recently synthesized via a non-aqueous fluorolytic sol-gel route. They contain a nominal fluoride concentration of 3,400 ppm, but solely dissolve low fluoride concentrations in aqueous solutions (n-CaF_2_: 7 ppm, n-MgF_2_: 70 ppm). For comparison, we simultaneously investigated an equally concentrated sodium fluoride solution, a marketed fluoride containing varnish (Duraphat) and pure ethanol. We hypothesized that these nanoscaled metal fluorides show similar remineralization of artificial caries lesions than the other modes of fluoride application either used in clinical practice or with similar theoretical free-fluoride concentrations.

## Results

For SL baseline mineral loss (ΔZ_Baseline_) ranged from 4649 to 4795 vol% × μm and LD_Baseline_ was between 108 to 119 µm. DL presented a ΔZ_Baseline_ from 9091 to 9304 vol% × μm and LD_Baseline_ was between 197 to 207 µm (Table 1). Mineral loss as well as the lesion depth of SL and for DL did not differ significantly within both subgroups at baseline (p > 0.05; Kruskal-Wallis test + Bonferroni adjustment).

**Table 1:** Mineral loss and lesion depths before and after pH-cycling for shallow and deep enamel lesions

Shallow (SL)	n	Mineral loss [vol% × μm]			Lesion depth [µm]		
Group		ΔZ_Baseline_	ΔZ_Effect_	p value	LD_Baseline_	LD_Effect_	p value
NaF	**16**	4720 (4488/4964)	3625 (2653/4159)	<0.005	119 (125/107)	90 (105/84)	0.01
n-CaF_2_	**16**	4795 (4656/5008)	3785 (3405/4171)	<0.005	116 (135/111)	112 (129/88)	0.999
n-MgF_2_	**18**	4742 (4621/5130)	3654 (3223/3997)	<0.005	117 (130/107)	90 (94/82)	<0.005
Duraphat	**18**	4649 (4549/5010)	3919 (3467/4438)	<0.005	110 (119/106)	102 (109/90)	0.175
Negative control	**18**	4723 (4428/5057)	4661 (4301/5464)	0.955	108 (121/104)	120 (136/107)	0.999
**Deep (DL)**		**Mineral loss [vol%** × **μm]**					
**Group**		**ΔZ**_**Baseline**_	**ΔZ**_**Effect**_	**p value**	**LD**_**Baseline**_	**LD**_**Effect**_	**p value**
NaF	**27**	9146 (8618/9656)	3808 (2750/5213)	<0.005	199 (188/227)	121 (104/152)	<0.005
n-CaF_2_	**28**	9145 (8616/10089)	3099 (2006/3801)	<0.005	204 (177/227)	130 (111/148)	<0.005
n-MgF_2_	**29**	9304 (8684/10108)	2304 (1771/2877)	<0.005	204 (177/215)	124 (100/134)	<0.005
Duraphat	**29**	9083 (8585/9628)	5446 (447/6721)	<0.005	197 (172/217)	148 (110/169)	<0.005
Negative control	**29**	9158 (8678/9729)	5476 (4655/6410)	<0.005	201 (183/221)	149 (137/178)	<0.005

Medians (25./75.percentiles) of mineral losses and lesions depths for specimens after pre-demineralization (ΔZ_Baseline_/LD_Baseline_) and after pH-cycling (ΔZ_Effect_/LD_Effect_). P values are given for pairwise comparison before and after treatment with the various agents (Wilcoxon test, Bonferroni adjusted). No significant differences were found for baseline values between groups (Kruskal-Wallis-test, Bonferroni adjusted); n = number sample size per group.

**Figure 1:**
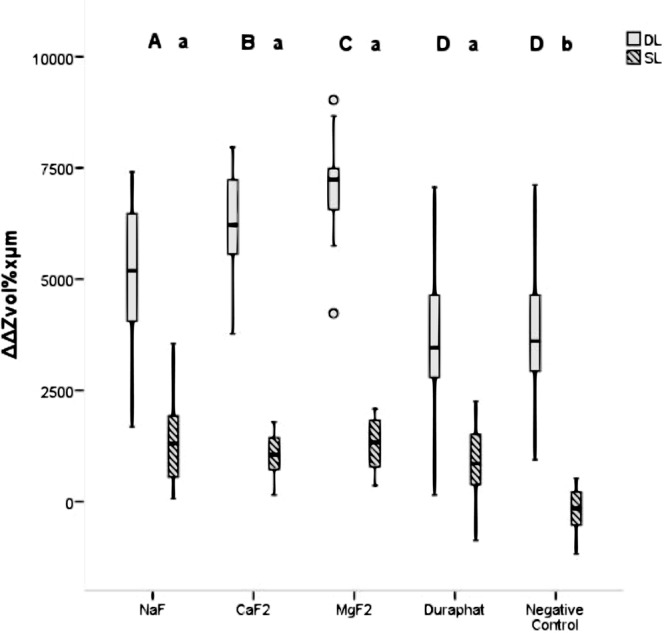
Mineral loss of shallow (SL) and deep (DL) artificial enamel caries lesions (ΔΔZ; box and thick lines = 25^th^ and 75^th^ percentiles and median, error bars = minima and maxima) after treatment with nanoscaled metal fluorides (n-CaF_2_: 7 ppm, n-MgF_2_: 70 ppm), an aqueous sodium fluoride solution (NaF: 3,400 ppm), a sodium fluoride containing varnish (Duraphat: 22,600 ppm) and a fluoride-free negative control (p < 0.05; Mann-Whitney test, Bonferroni adjusted); circles = outliners. Different upper-case letters indicate statistically significant difference between DL and lower-case letters between SL groups. Different upper-case letters indicate statistically significant difference between DL and lower-case letters between SL groups.

**Figure 2:**
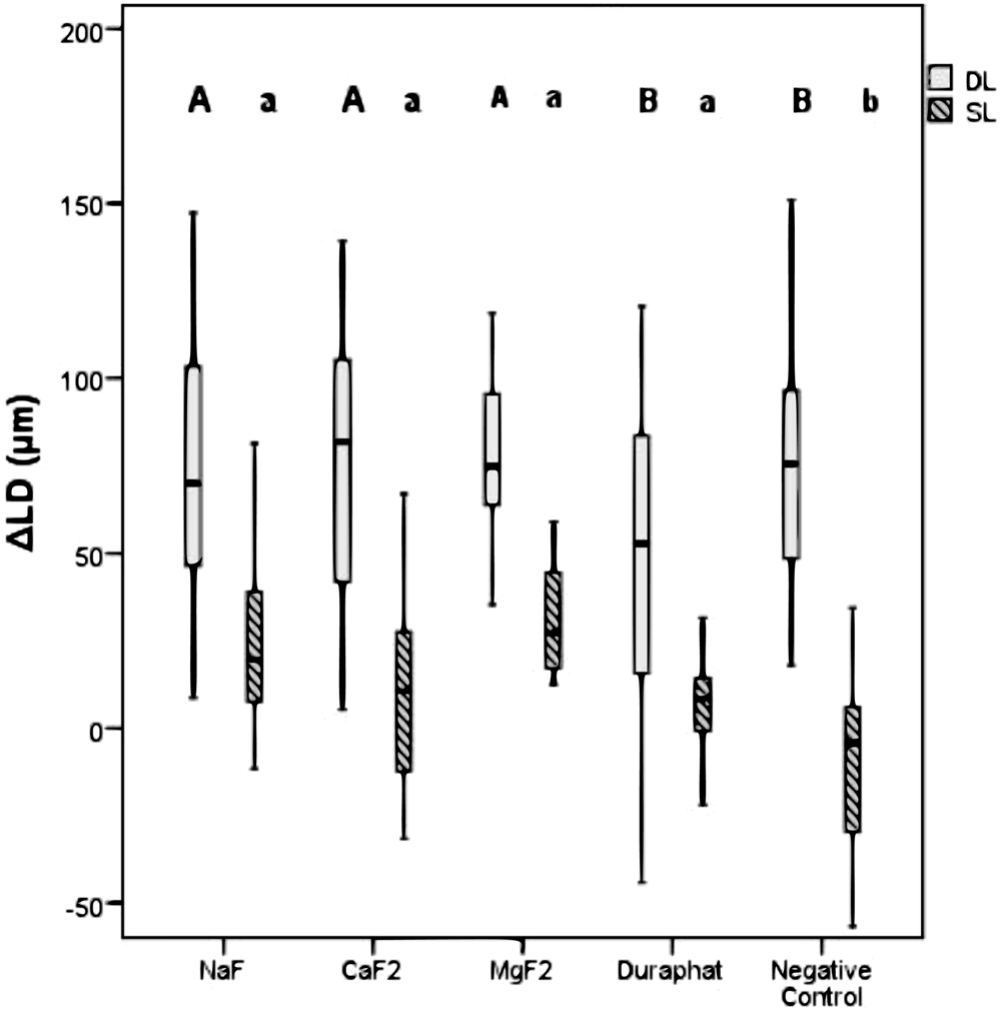
Lesion depth of shallow (SL) and deep (DL) artificial enamel caries lesions (ΔLD; box and thick lines = 25^th^ and 75^th^ percentiles and median, error bars = minima and maxima) after treatment and pH-cycling with nanoscaled metal fluorides (n-CaF_2_: 7 ppm, n-MgF_2_: 70 ppm) compared with an aqueous sodium fluoride solution (NaF: 3,400 ppm), a sodium fluoride containing varnish (Duraphat: 22.600 ppm) and a fluoride-free negative control were investigated (p < 0.05; Mann-Whitney test, Bonferroni adjusted); circles = outliners. Different upper-case letters indicate statistically significant difference between DL and lower-case letters between SL groups. Different upper-case letters indicate statistically significant difference between DL and lower-case letters between SL groups.

SL specimens treated with NaF, n-CaF_2_, n-MgF_2_ and Duraphat showed a significant remineralization (ΔZ_Effect_) compared to ΔZ_Baseline_ (p < 0.05; Wilcoxon test + Bonferroni adjustment; Table 1), which was not the case for the negative control (p > 0.05, Table 1). All fluoride groups showed significantly higher remineralization (ΔΔZ and ΔLD) compared with the negative control (p < 0.05; Mann-Whitney test + Bonferroni adjustment; Figs 1 and 2) but did not differ significantly amongst each other (p > 0.05).

For DL, all groups showed a significant remineralization in ΔZ_Effect_ compared to ΔZ_Baseline_ and reduced lesion depths (LD_Effect_) compared with baseline (LD_Baseline_) (p < 0.05; Wilcoxon test + Bonferroni adjustment; Table 1). DL that were treated with n-CaF_2_ and n-MgF_2_ showed significantly higher remineralization (ΔΔZ) compared to treatments with NaF, Duraphat or the negative control group after 90 days of pH-cycling (p < 0.05; Mann-Whitney test + Bonferroni adjustment; Fig. 1). The negative control group and Duraphat revealed significantly lower lesion depth changes (ΔLD) indicating less remineralization compared to specimens treated with NaF, n-MgF_2_ or n-CaF_2._ (p < 0.05; Mann-Whitney test + Bonferroni adjustment; Fig. 2).

For bioavailable fluoride measurements for NaF and Duraphat similar results in comparison to their theoretically calculated values in aqueous solution could be revealed. The ethanol-based n-CaF_2_ and n-MgF_2_ showed lower results as their theoretically calculated values for application in water. Data for n-CaF_2_ and the negative control presented barely detectable concentrations of bioavailable fluoride (Table 2).

**Table 2:** Measurements conducted with the fluoride sensitive electrode.

Sample	Concentration of bio- available F- in mol/L	Concentration of bio-available fluoride in g/L	Concentration of bio-available fluoride in ppm
NaF in water	1.98 × 10^−1^	3.76	3760
CaF_2_ sol	1.8 × 10^−5^	0.3 × 10^−3^	0.3
MgF_2_ sol	3.8 × 10^−4^	7.2 × 10^−3^	7.2
Duraphat	1.05	19.95	19950
negative control	1.8 × 10^−5^	0.34 × 10^−3^	0.34

The fluoride bioavailable concentrations of the applied test solutions are displayed in the table.

## Discussion

The results of the present study indicate that nanoscaled metal fluorides, which dissolve low fluoride concentrations in aqueous solutions, promote remineralization of enamel caries lesions to a similar extent compared with a 3,400 ppm F^−^ sodium fluoride solution or Duraphat after a short-term period of 14 days and present a significant greater ability to remineralize enamel caries lesions after an extended period of 90 days. Thus, the hypothesis of similar remineralization abilities of the nanoscaled metal fluorides compared with a 3,400 ppm F^−^ sodium fluoride solution or a commonly applied varnish (Duraphat) could be confirmed for shallow enamel caries lesions but must be rejected for deep enamel caries lesions.

In this study the NMF contained an equal nominal fluoride concentration as the investigated sodium fluoride solution. In aqueous solutions the NMF dissolve very low concentrations of free fluorides (n-CaF_2_: 7 ppm; n-MgF_2_: 70 ppm) in comparison to the tested sodium fluoride solution (NaF: 3400 ppm F^−^). In theory, the sodium fluoride solution releases 24 times (NaF vs. n-MgF_2_) or even 242 times (NaF vs. n-CaF_2_) more free fluoride for remineralization. The results of the FSE confirmed an even greater difference of free fluoride concentrations between the NMF and the sodium fluoride applications (NaF:3400 ppm, Duraphat). Based on our results, the influence of fluoride application not merely depends on the fluoride concentration. The presence of free fluorides is a crucial point for remineralization^23^. The dose/retention rate may vary significantly depending on the applied forms and modes of application^24^. The two separately conducted pH-cycling models revealed at least a similar remineralization potential for the NMF as observed for the other fluoride modes, despite their low concentrations of free fluorides in aqueous solutions. In an earlier investigation, nanoscaled calcium fluoride formation have already proven to release seven times more fluorides for the remineralization process than macroscaled fluoride modes of the same nominal concentration^20^.

The tested NMF are small in size but have a large surface ranging from 229 m^2^ g^−1^ ± 64 m^2^ g^−1^ to even more than 300 m^2^ g^−1^ and possess a great level of Lewis acidity^25–27^. Therefore, these metal fluorides present a higher reactivity than their macroscaled chemical equivalences. Thus, the dissolved low free fluoride concentrations of the investigated NMF seem to be capable of contributing equally to the remineralization process as a 3,400 ppm F^−^ sodium fluoride solution. Free fluoride concentrations as little as 1 ppm in an acid solution may reduce the solubility of enamel, furthermore low free concentrations present a greater effect in the reduction of the solubility of enamel as structurally bounded fluoride does^28^. Similarly, the low fluoride concentrations of the tested NMF can enhance remineralisation of enamel lesions significantly.

Furthermore, based on the results obtained here, it can be evidently deduced that – corresponding to our working hypothesis – the heavily soluble nanoscaled metal fluorides diffuse into the caries areas of the specimens, thus forming a depot from which fluoride ions slowly become released controlled by the low solubility of n-MgF_2_ and n-CaF_2_, respectively.

Previous studies emphasized the importance of a cariostatic concentration of F^−^ in oral fluids. The tooth surface acts as a mineral reservoir of calcium fluoride or ‘calcium-fluoride-like’ deposits as well as the dental plaque serving as a biological reservoir^10,29,30^. Deposition of fluoride releasing nanoparticles in dental resins on the lesion’s surface have been studied, but not the abilities of nanoscaled fluorides infiltrated within the lesion itself^31^. The enamel’s pore volume increases during a cariogenic acid attack. Consequently, it becomes more accessible for ions to permeate into the enamel structure^12,28^. The tested NMF are smaller than <15 nm, the reduced size enables the NMF to penetrate the enamel matrix and to form a reservoir within the enamel lesion^27^. Thus, a constant contribution to the enamel’s remineralization process is possible. This seems to be reflected by the significantly higher remineralization of DL after 90 days of pH-cycling by the NMF compared to the other agents.

A reduction of mineral loss as well as lesion depths could be obtained by all kinds of fluoride applications in SL. After 90 experimental days (DL), NMF presented significantly greater mineral gain and reduction of lesion depth compared with the tested highly fluoride-containing varnish (Duraphat). This difference between fluoride agents as well as pH-cycling periods might be explained by early clogging of the enamel pores^32^.

Notably for fluoride varnishes the extent of remineralization is independent from its direct location. Varnishes applied on or around the caries lesion present similar results^33^. In both experimental settings the varnish was directly applied on the tested SL or DL and softly removed after 48 h. The removal was challenging due to the resin characteristic adherence to the enamel. This might have contributed to a pore clogging effect, as well. Furthermore, in experiments with fluoride varnishes, structurally bound fluoride was detected as early as one day after application at the direct treatment site, where it has been incorporated into the enamel crystallites^10,29^. The calcium like formations generated by fluoride varnishes mainly remained on the surface^10,34,35^. At the surface plugging of enamel pores and interprismatic areas by calcium fluoride formations hinders diffusion of further ions into the lesion. Thus, remineralization by fluorides from Duraphat could have been limited to superficial enamel only. Notably, clogging of enamel pores may inhibit further dissolution and subsequent demineralization of enamel. Early caries lesion in enamel possesses a pore size of 50–200 nm, the investigated NMF are similarly produced as the metal fluorides, with a size between 10–15 nm^27,36^. Therefore, these NMF should easily penetrate the enamel structure without clogging of superficial pores due to their limited amount of free reactive fluoride ions and thus can provide a fluoride reservoir within the lesion.

Enamel apatite is highly crystalline with a thin film of firmly bound water surrounding the crystals. The presence of water is associated with the porosity of the tissue^4^. Synthetic nano-hydroxyapatites in sizes of about 20 nm are able to penetrate into the porous lesion body^4^. Hereby, they prevent secondary caries formation and restore its hardness^37^. Larger scaled nano-hydroxyapatites of hundreds of nanometres are considered to reform enamel *in vitro*. Unfortunately, this concept of rebuilding enamel *in vivo* fails^37^.

Dimension, morphology and orientation of the native enamel structure seem to be too complex to be rebuilt by synthetic nano-rods *in vivo*. The NMF of the present study are constructed by fluorolytic sol gel route and consist out of magnesium fluoride or calcium fluoride. Thus, they are not conceptualized to rebuild the natural structure, but they might ensure remineralization without clogging. Furthermore, the n-CaF_2_ provides, besides fluoride, calcium for remineralization of enamel caries lesions for a possibly improved inhibition of demineralization^38^. Unlike macroscaled calcium fluoride formations, which are known to be KOH-soluble and can be leached away with a few days^35^. The nanoscaled calcium formations possess a low aqueous solubility, which possibly substantiates their stability on and in the enamel lesion, as well^26^. Notably, the dissolution of n-MgF_2_ sets free magnesium ions. Incorporation of magnesium ions into the hydroxyapatite structure results in higher solubility of the hydroxyapatite structure in case of a cariogenic environment^2^. Nevertheless, the nanoscaled magnesium fluoride delivered similar remineralization in both pH-cyclings as n-CaF_2_.

The results of this *in vitro* study cannot be transferred simply to the clinical application. For some nanomaterials, application requires either an acidic milieu, high temperature, high pressures or the presence of amelogenin to form chemically and structurally resembled enamel from nanoscaled hydroxyapatite crystals^4,13^. Thus, formation of newly-formed hydroxyapatite crystals by the tested NMF is unlikely.

Nanoscaled metal fluorides were softly brushed on the enamel surface and softly dried with compressed air. This application form appears to be feasible *in vivo*. However, the usage of a rubberdam might be necessary to isolate the teeth from the oral environment^39^.

### Conclusion

The investigated NMF have the ability to remineralize early enamel caries lesions *in vitro* after a rather short-term period of 14 days of pH-cycling and might show enhanced remineralization abilities compared with 3,400 ppm F^−^ sodium fluoride solution or varnish application (Duraphat) after a long-term period of 90 days.

## Methods

### Sample Preparation

Bovine incisors were obtained from cattle slaughtered in the food manufacturing industry (negative BSE test, concentration of fluoride in drinking water was <0.2 mg/l) and stored in 0.08% thymol. Stained or cracked teeth with structural defects were excluded. Teeth were carefully cleaned and a total of 231 samples were prepared from the labial areas (6 × 4 × 4 mm) under running tap water using a diamond coated band saw (Trennschleifsystem 300 cl; Exact, Norderstedt, Germany). After sterilization with ethylene dioxide, the enamel blocks were embedded in epoxy resin (Technovit 4071; Heraeus Kulzer, Wehrheim, Germany). All embedded samples were ground flat and polished (Mikroschleifsystem 400 cs; Abrasive Paper 800, 1200, 2400, 4000, Exact Apparatebau). Specimens were partially covered with acid-proofed nail varnish (sound control). The other half (treatment area) remained uncovered and was subsequently demineralized. Therefore, specimens were stored in a demineralization solution for either 14 (n = 86, SL) or 21 days (n = 145, DL) at 38 °C (0.6 μM methylhydroxydiphosphonate, 3 mM CaCl_2_, 3 mM KH_2_PO_4_ and 50 mM acetic acid adjusted to pH 4.95, as described previously^40^). All specimens developed subsurface lesions consistently revealing a surface area that was more mineralized than the body of the lesion, with no signs of erosive loss or destruction of the lesion surface. After demineralization thin plano-paralell slices of 100 µm were cut and grinded perpendicular to the lesion surface for transversal microradiographical analysis (TMR).

### Division of Groups and Tested Fluoride Applications

Specimens were randomly allocated to five groups, which received different fluoride treatments; one served as negative control (pure ethanol).

The NMF were recently synthesized via a non-aqueous fluorolytic sol-gel route^25,26^. These tested NMF contain a nominal fluoride concentration of 3,400 ppm, but solely dissolve low fluoride concentrations in aqueous solutions (n-CaF_2_: 7 ppm, n-MgF_2_: 70 ppm). For comparison, we simultaneously investigated an equally concentrated sodium fluoride solution (NaF: 3,400 ppm), a marketed sodium fluoride containing varnish (Duraphat). The two nanoscaled metal fluorides were prepared by a non-aqueous fluorolytic sol-gel route.

### Determination of free fluoride concentration in the test solutions

All tested solutions (n-CaF_2_, n-MgF_2_, NaF) were applied as an aqueous phase. Only free fluoride ions may have contributed to remineralization. Thus, the concentration of free fluoride ions was calculated with respect to the individual solubility products of the different fluoride modes.

Solubility of the three fluorides in water:

$$\begin{array}{rcl}{{\rm{L}}}_{{\rm{CaF2}}} & = & 0,16\,{\rm{g}}/{\rm{L}}= > 0,4\times {{\rm{10}}}^{-{\rm{3}}}\,{\rm{mol}}\,{{\rm{F}}}^{-}/{\rm{L}}\\ {{\rm{L}}}_{{\rm{MgF2}}} & = & 0,13\,{\rm{g}}/{\rm{L}}= > 4,0\times {{\rm{10}}}^{-{\rm{3}}}\,{\rm{mol}}\,{{\rm{F}}}^{-}/{\rm{L}}\\ {{\rm{L}}}_{{\rm{NaF}}} & = & 8\,{\rm{g}}/{\rm{L}}= > {\rm{190}}\times {{\rm{10}}}^{-{\rm{3}}}\,{\rm{mol}}\,{{\rm{F}}}^{-}/{\rm{L}}\end{array}$$

The calculation was based on one kilogram of water (about 55.5 mol), in which the maximum amounts of free fluoride are present. The individual solubility of free fluoride from the different fluoride modes are set in proportion to one kilo of water, thus their maximum possible bioavailability can be calculated:

$$\begin{array}{rcl}{\rm{CaF2}} & = & 0,4\times {10}^{-3}\,{\rm{mol}}\,{{\rm{F}}}^{-}/\mathrm{55},5\,{\rm{mol}}\,{\rm{H2O}}\,\approx \,{\rm{7}}\,{\rm{ppm}}\,{{\rm{F}}}^{-}\\ {\rm{MgF2}} & = & 4,0\times {{\rm{10}}}^{-{\rm{3}}}\,{\rm{mol}}\,{{\rm{F}}}^{-}/\mathrm{55},5\,{\rm{mol}}\,{\rm{H2O}}\,\approx \,{\rm{70}}\,{\rm{ppm}}\,{{\rm{F}}}^{-}\\ {\rm{NaF}} & = & {\rm{190}}\times {{\rm{10}}}^{-{\rm{3}}}\,{\rm{mol}}\,{{\rm{F}}}^{-}/\mathrm{55},5\,{\rm{mol}}\,{\rm{H2O}}\,\approx \,{\rm{3400}}\,{\rm{ppm}}\,{{\rm{F}}}^{-}\end{array}$$

In addition, the F-ion concentration was determined by a potentiometric method with a fluoride sensitive electrode (Table 2). The fluoride sensitive electrode was calibrated for direct ion measurement by determining the measured potential in mV for various known sodium fluoride concentrations under equal conditions by applying a pH- and ion conductivity buffer as described in literature.

### Surface treatments and pH-cycling

Before surface treatment and pH-cycling, sound control areas of the enamel specimens were sealed with a light curing composite (TetricEvoFlow; IvoclarVivadent, Schaan, Lichtenstein) to prevent the alcoholic based test solutions from influencing the control site of demineralization. The respective fluoride solutions were applied using applicator tips. Afterwards, compressed air was used to softly dry the solutions on the enamel lesions for approximately 60 s. Hereby, dispersal of the fluoride solutions was avoided. This process was repeated five times with 20 s drying in-between for each fluoride solution in each group as well as for the negative control. Duraphat varnish was applied and removed after 48 h by soft rubbing using a cotton pellet.

After the treatments a pH-cycling was conducted for either 14 days (SL) or 90 days (DL). Demineralizing and remineralizing solutions were prepared as described previously^40^. The remineralization solutions contained 1.5 mM CaCl_2_, 0.9 mM KH_2_PO_4_ and 20 mM Hepes at pH 7.0. The demineralization solution contained 0.6 µm methylhydroxydiphosphonate, 3 mM CaCl_2_, 3 mM KH_2_PO_4_ and 50 mM acetic acid adjusted to pH 4.95^40^. The pH-cycling solutions were refreshed with each cycle. Each specimen was cycled in 25-ml aliquots of the solutions. Thus, the amounts of each solution were large enough to prevent the solutions from becoming saturated with or depleted of mineral ions.

pH-cycling conditions were chosen with a daily schedule of four cycles, hereby each one-hour demineralizing phases (pH 4.95, t = 4 h/24 h) was framed by intermediate remineralizing phases (pH 7.0, t = 20 h/24 h). Specimens were washed with deionized water in-between phases for a minimum of 60 s.

### Transversal Microradiography

After the pH-cycling thin plano-parallel sections were prepared again (100 µm). Microradiographs of the enamel specimens were obtained using a nickel-filtered copper (CuKa) X-ray source (PW 1730; Phillips, Kassel Germany) and analysed as described previously^41^. The investigator was blinded with respect to the group allocation. The mineral content was calculated by TMR software (Version 5.25 by Joop de Vries, Groningen, Netherlands) depending on the gray levels of the specimens. Profiles of mineral density were evaluated and integrated mineral loss (ΔZ) and lesion depth (LD) were calculated after the baseline demineralization (Δ*Z*_Baseline,_ LD_Baseline_) as well as after the treatment with the various solutions or the varnish after the pH-cycling (Δ*Z*_Effect,_ LD_Effect_). Changes in mineral loss (ΔΔ*Z* = Δ*Z*_Baseline_ − Δ*Z*_Effect_) and lesion depth (ΔLD = LD_Baseline_ − LD_Effect_) were calculated^41^. Positive and negative values of ΔΔ*Z* indicated net remineralization or net demineralization, respectively.

### Statistical analysis

Data were analysed using SPSS statistical software (SPSS Version 20.0; IBM, Munich, Germany). All variables were tested for normal distribution using the Shapiro-Wilk test. Difference in ΔZ and LD before and after pH-cycling were analysed for related samples with the non-parametric Wilcoxon signed-rank test. For both pH-cycling durations, ΔΔZ and ΔLD of groups were compared using Kruskal-Wallis and Mann-Whitney test. All tests were performed at a 5% level of significance. Bonferroni adjustment was used to counteract the problem of multiple comparisons, p-values were adjusted accordingly.

### Ethical approval and informed consent

The experiments were carried out in accordance with the relevant guidelines and regulations of the the RWTH Aachen University and the Christian-Albrechts-Universität zu Kiel, Germany. All bovine teeth were obtained from cattle slaughtered in the food manufacturing industry. No animals were exclusively harm in order to conduct this study.

## Data Availability

We agree to make materials, data and associated protocols promptly available to readers without undue qualifications in material transfer agreements.
